# Detection limitations of prion seeding activities in blood samples from patients with sporadic prion disease

**DOI:** 10.1186/s12883-024-03590-7

**Published:** 2024-03-11

**Authors:** Toshiaki Nonaka, Yasushi Iwasaki, Hiroyuki Horiuchi, Katsuya Satoh

**Affiliations:** 1 Division of Cellular and Molecular Biology, Nagasaki University Graduate School of Biomedical Sciences, Nagasaki City, 852-8501 Japan; 2 Department of Neuropathology, Institute for Medical Science of Aging, Aichi Medical University, Nagakute City, 480-1195 Japan; 3 Laboratory of Immunobiology, Graduate School of Integrated Sciences for Life, Hiroshima University, Higashihiroshima City, Japan; 4 Department of Health Sciences, Unit of Medical and Dental Sciences, Nagasaki University Graduate School of Biomedical Sciences, Nagasaki City, Japan

**Keywords:** Abnormal prion protein, eQuIC, Sporadic prion disease, Prion seeding activity, Diagnostic technique, blood

## Abstract

**Background:**

Human prion diseases (HPDs) are fatal neurodegenerative disorders characterized by abnormal prion proteins (PrPSc). However, the detection of prion seeding activity in patients with high sensitivity remains challenging. Even though real-time quaking-induced conversion (RT-QuIC) assay is suitable for detecting prion seeding activity in a variety of specimens, it shows lower accuracy when whole blood, blood plasma, and blood-contaminated tissue samples are used. In this study, we developed a novel technology for the in vitro amplification of abnormal prion proteins in HPD to the end of enabling their detection with high sensitivity known as the enhanced quaking-induced conversion (eQuIC) assay.

**Methods:**

Three antibodies were used to develop the novel eQUIC method. Thereafter, SD50 seed activity was analyzed using brain tissue samples from patients with prion disease using the conventional RT-QUIC assay and the novel eQUIC assay. In addition, blood samples from six patients with solitary prion disease were analyzed using the novel eQuIC assay.

**Results:**

The eQuIC assay, involving the use of three types of human monoclonal antibodies, showed approximately 1000-fold higher sensitivity than the original RT-QuIC assay. However, when this assay was used to analyze blood samples from six patients with sporadic human prion disease, no prion activity was detected.

**Conclusion:**

The detection of prion seeding activity in blood samples from patients with sporadic prion disease remains challenging. Thus, the development of alternative methods other than RT-QuIC and eQuIC will be necessary for future research.

**Supplementary Information:**

The online version contains supplementary material available at 10.1186/s12883-024-03590-7.

## Introduction

Human prion diseases (HPDs) or transmissible spongiform encephalopathies (TSEs) are fatal transmissible neurodegenerative diseases in humans [1] and are classified into sporadic, genetic, and acquired forms, the most common being the sporadic form of the Creutzfeldt–Jakob disease (sCJD). The annual incidence rate of sCJD is 1.4 per million people in Japan [2]. Genetic prion disease is characterized by causal mutations in the human prion protein gene (PRNP) or a relevant family history, including Gerstmann–Sträussler–Scheinker disease, fatal familial insomnia (FFI), or genetic CJD (gCJD). The acquired forms are dura-associated CJD (dCJD) and variant CJD derived from bovine spongiform encephalopathy [3].

The infectious agent, or prion, of TSEs appears to be composed of an abnormal prion protein (PrPSc). PrPSc is formed post-translationally from the normal cellular prion protein (PrPc), which in purified form can resemble amyloid fibrils. PrPSc induces the polymerization and conformational conversion of PrPc into infectious PrPSc or PrPSc-like partially protease-resistant PrP forms in a variety of in vitro reactions [1].

In vitro techniques for amplifying PrPSc, such as protein misfolding cyclic amplification (PMCA) [4], amyloid seeding assay (ASA) [5], and quaking-induced conversion (QuIC) [6], enable the highly sensitive detection of PrPSc. QuIC assays use recombinant normal prion protein as a substrate to amplify minute quantities of PrPSc with intermittent automated shaking, which can be performed more easily than the PMCA-associated sonication. We recently developed a novel in vitro amplification technology, designated as the “real-time quaking-induced conversion (RT-QuIC)” assay, which enables the detection of prion seeding activity in various organ tissues derived from patients with sCJD [7]. Previously, we developed the RT-QuIC assay primarily for identifying minute quantities of abnormal PrP within tissue and body fluids. We demonstrated that the RT-QuIC assay is more sensitive than bioassays. Notably, the end-point RT-QuIC assay can be used to evaluate human prion seeding activity (PSA) in the brains of patients with HPDs [8]. Given the recent advancements in drug development for prion diseases and the initiation of clinical trials, a growing need exists to measure prion activity in non-invasive tissues, such as blood. However, detecting PSA in blood poses considerable challenges. Therefore, to circumvent this issue and move toward developing a blood test for prions, we introduced an immunoprecipitation step at the beginning of the RT-QuIC reaction. We further enhanced its sensitivity through the addition of an rPrP substrate replacement step during the RT-QuIC reaction, an approach previously shown to be helpful in PMCA reactions [4]. The combination of these two assay modifications resulted in an enhanced method that we termed “eQuIC” [9].

In a previous study, the target animals of the “eQuIC” method were mice and humans, but the target disease in humans was variant CJD [10]. Notably, the “eQuIC” method is capable of detecting prion seeding activity in blood samples of patients with variant CJD. However, the detection of prion seeding activity in the blood samples of patients with sporadic HPD (sHPD) could not be achieved. In this study, we aimed to improve the “eQuIC” method for detecting prion seeding activities in the blood samples of patients with sHPD.

## Materials and methods

### Sample collection and preparation

For our analysis, we used endpoint RT-QuIC assays to examine brain samples obtained from three confirmed cases of sHPDs (specifically of the MM1 subtype, according to the Parchi classification [2]; the cases correspond to patients #1, #2, and #3). We included two non-CJD brain specimens as negative controls. The brain tissue samples were homogenized in 10% (w/v) ice-cold phosphate-buffered saline (PBS) supplemented with a protease inhibitor mixture (Roche, Mannheim, Germany) using a multi-bead shocker (Yasui Kikai, Osaka, Japan). Brain homogenates (BHs) (10% [w/v]) were serially diluted (10-fold) using a material solution containing 125 mM NaCl, 2.5 mM KCl, 2 mM CaCl2, 1 mM MgCl2, 0.2 ng/mL BSA, and 0.05% glucose. Thereafter, the samples were centrifuged at 6000 rpm (10,000 g) and room temperature (15–20 °C) for 2 min to clarify the supernatant. The clarified samples were stored at − 80 °C until further use.

We collected blood samples (total blood, serum, and plasma) from patients #1–3 within one month of onset of disease (Table 1). The Ethics Committee, Nagasaki University Graduate School of Biomedical Sciences, endorsed the study protocol (ID Nos. UMIN000038398, UMIN000016855, and UMIN000003301). " The Ethics Committee, Nagasaki University Graduate School of Biomedical Sciences, approved the study protocol. All patients or family members of patients participating in this research have understood and agreed to the ethics committee documents for this research. The research was conducted in accordance with the Declaration of Helsinki and the “Ethical Guidelines for Medical Research Involving Human Subjects” issued by the Ministry of Health, Labor and Welfare. T The Ethics Committee, Nagasaki University Graduate School of Biomedical Sciences. research.

**Table 1:** Summary of patients with sporadic human prion disease

Patient number	1	2	3
Sex	Female	Female	Male
Age at onset (years)	69	74	73
Time from onset till death (months)	27	4	16
sCJD subtype based on Parchi’s classification	MM1	MM1	MM1
log SD_50_/g tissue in brains homogenates based on the RT-QuIC assay (mean ± SD)	10.38 ± 0.18	10.25 ± 0.35	10.00 ± 0.00
log SD_50_/g tissue in brains homogenates based on the second generation QuIC assay (mean ± SD)	10.50 ± 0.50	10.75 ± 1.15	10.60 ± 1.25
log SD_50_/g tissue in brains homogenates based on the eQuIC assay (mean ± SD)	12.75 ± 0.35	12.25 ± 0.18	12.50 ± 0.35

### RT-QuIC, endpoint RT-QuIC, and second-generation RT-QuIC assay

Analyses of the RT-QuIC and endpoint RT-QuIC assays were similar to the previously described methodology for unfixed brain RT-QuIC assays [7]. In brief, the RT-QuIC reaction mix consisted of 50 mM PIPES (pH 7.0), 500 mM NaCl, 10 µM thioflavin-T (ThT), 0.1 mM ethylenediaminetetraacetic acid tetrasodium salt hydrate (EDTA), and recombinant human PrP (residues 23–231 with 129 M). Each well of a black 96-well plate with a clear bottom (Nunc 96 well; Sigma-Aldrich, St. Louis, MO, USA) was loaded with 90-µL aliquots of the reaction mix and 10 µL of brain homogenate. Four to eight replicates of each diluted sample were assessed, and PrP amyloid formation was monitored for 48 h. We calculated the 50% seeding dose (SD50) in unfixed brains, fixed brains, and formic acid-treated brains using the Spearman–Kärber method [8].

We used the RT-QuIC assay and defined a reaction as positive when the following three conditions were met: (1) the absorbance exhibited an initial rise within 150 cycles, maintaining a stable level after attaining the maximum absorbance; (2) the maximum absorbance was > 6-fold higher than the initial absorbance; and (3) only recombinant protein was used to maintain the quality of the batch, provided that the prepared recombinant protein displayed an SD50 ≥ 10^7^, as determined using the RT-QuIC assay in the MM1-type sCJD brain.

Alternatively, the second-generation RT-QuIC assay was performed as previously described [11]. The assay was repeated at least twice, and Spearman–Kärber analysis was used to estimate the SD50. The SD50 was calculated using the following formula: xp = 1 + 1/2d − dgp, where xp = 1 represents the highest log dilution that generates all positive responses, d represents the log dilution factor, p denotes the proportion positive at a given dose, and gp denotes the sum of p values for xp = 1 and all higher dilutions [9].

After purification, rHuPrP was stored at − 80 °C. Brain homogenates (BHs) (10% [w/v]) were serially diluted (10-fold) with a material solution containing 125 mM NaCl, 2.5 mM KCl, 2 mM CaCl2, 1 mM MgCl2, 0.2 ng/mL BSA, and 0.05% glucose. Each well of a 96-well plate was loaded with rHuPrP suspended in 90 µL of RT-QuIC buffer (500 mM NaCl, 50 mM PIPES (pH 7.0), 10 µM ThT, and 1 mM EDTA), and mixed with 10 µL of the brain samples or blood samples. The assay was monitored for 40 h. Four to eight replicates of each diluted sample were measured. The SD50 was calculated using the Spearman–Kärber method. The same organs derived from two healthy individuals were used as negative controls in all RT-QuIC assay experiments using the remaining eight wells.

### Antibody (3F4, 3S9, and 2H9) coating of magnetic beads

3F4 antibody is already the most used antibody by many prion researchers, and we collaborated with Hiroshima University to create two antibodies (3S9 and 2H9) to use in this experiment. These two antibodies recognize the C-terminus of the PRNP gene region 110–231. The antibodies (3F4, 3S9, and 2H9) were identified in the brains of the patients with sHPD. We outsourced the general procedure of attaching the antibodies to magnetic beads to Tamagawa Seiki Kaihatsu Shikkai Co. (Nagano prefecture, Chuubu area, Japan). Mock control beads were prepared as described for the 3F4, 3S9, and 2H9 antibody-coated beads, but without the addition of these antibodies.

### Preparation of magna bind beads

MagnaBind beads (Pierce, Rockford, IL, USA) were vortexed for 30 s, and 1.2 × 108 beads were transferred to a fresh tube. The beads were rinsed twice with 500 µL of 0.5% Triton X-100 in PBS and resuspended in their initial volume using assay buffer (Tris-buffered saline, 1% Triton X-100, and 1% Tween 20).

### eQuIC assay on blood (serum, plasma, and total blood) and brain homogenates from patients with sHPD

The antibody-coated beads, mock beads, and Magna Bind beads were briefly vortexed, and 2 × 107 beads were transferred to a fresh tube. A volume of 500 µL of human blood (serum, plasma, and total blood) was added to the beads. Following 2 min of incubation on the magnet, the storage (coating) buffer was discarded, and 500 µL of immunoprecipitation buffer (Prionics) was added. Thereafter, 500 µL of BH-spiked human plasma or 500 µL of plasma or the brain homogenate from patients with HPD was added to the beads. The samples were incubated with “end-over-end” rotation at room temperature or 37 °C overnight (ON). Subsequently, the samples were incubated on the magnet for 2 min, plasma-buffer mix was discarded, and beads were washed twice with 500 µL of wash buffer. All beads were then resuspended in 10 µL of PBS and used immediately.

## Results

### RT-QuIC and endpoint RT-QuIC, eQuIC and second-generation RT-QuIC assays in brain homogenate from HPD patients

**Fig. 1:**
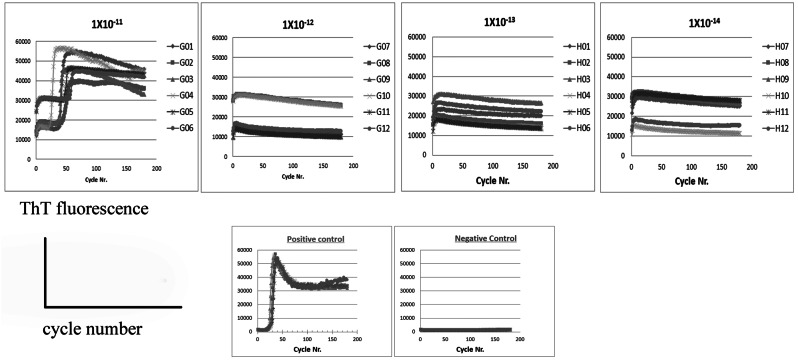
Analysis of brain tissues from patients with sporadic human prion disease using bioassay and endpoint RT-eQuIC assay. Endpoint RT-eQuIC assay of brain tissue from Patient #1

Three patients with HPD were designated as the MM1 subtype based on Parchi classification. The log SD50 obtained from the RT-QuIC assay for these three patients (patients #1, #2, and #3) were 10.38 ± 0.18, 10.25 ± 0.35, and 10.00 ± 0.00, respectively. Similarly, using the second-generation QuIC assay, the log SD50 values for the patients were 10.50 ± 0.50, 10.75 ± 1.15, and 10.60 ± 1.25, respectively. The newly developed eQuIC assay in the HPD patients yielded SD50 values of 12.75 ± 0.35, 12.25 ± 0.18, and 12.50 ± 0.35, respectively (Table 1)(Fig. 1). On comparing the log SD50 values between the eQuIC and RT-QuIC methods, our results indicate that the eQuIC assay displays an approximate 2–2.5-fold higher sensitivity than the RT-QuIC method. Considering that this sensitivity increment translates to an approximate 100–300-fold enhancement in sensitivity, our method may have the potential to detect as little as ≧ 10 ag of PrPSc in brain homogenates derived from patients with HPD.

Given the possibility that this may be attributable to differences in the sensitivity of recombinant proteins, we investigated the differences in recombinant proteins between humans and hamsters. We also examined the performance of the second-generation RT-QuIC assay using recombinant proteins in hamsters (Supplementary Material and Table 1). Our results suggest that the RT-QuIC and second-generation RT-QuIC assays achieved similar SD50 values for recombinant proteins in hamsters (Supplementary Table 1).

### eQuIC Assay on blood samples from HPD patients

We performed approximately 1,000 different eQuIC assays on blood (plasma, serum, and total blood) samples from patients with HPD; however, we were unable to detect PSAs in the blood samples using the eQuIC assay (Table 2).

**Table 2:** Summary of sporadic human prion disease patient blood (serum, plasma, and total blood) analyzed using the eQuIC assay

Patient number	1	2	3
log SD_50_/g tissue in brains homogenates based on the eQUIC assay (mean ± SD)	12.75 ± 0.35	12.50 ± 0.18	12.15 ± 0.35
log SD_50_/g tissue in bloods based on the eQUIC assay	N.D.	N.D.	N.D.

N.D.: Not detected

## Discussion

We recently developed a novel in vitro amplification method, the eQuIC assay, which enabled the detection of ≥ 10 ag of PrPSc in diluted brain homogenates from patients with sHPD. The eQuIC assay was approximately 100 times more sensitive than the RT-QuIC assay at detecting PrPSc from brain homogenates of patients with sHPD.

Furthermore, Orrú et al. highlighted the potential of eQuIC for routinely detecting minute quantities of prion in tissues, fluids, and environmental samples [9, 10]. They used the eQuIC assay to detect prions in the plasma of patients with variant CJD and demonstrated that eQuIC could effectively distinguish between plasma samples from infected and uninfected hamsters and sheep with scrapie.

Furthermore, evidence points to the presence of infectious agents in the erythrocytes, leukocytes, and plasma of both variant and sporadic CJD patients [12, 13]. The measured infectivity levels were comparable to those reported in various prion disease animal models [8]. Using unconventional methods, we revealed the distinct MM2-cortical and thalamic subtypes of each patient’s CSF, unseen by standard techniques (data unshown).

However, our attempts to detect PSAs in the plasma, whole blood, and serum from sHPD patients were unsuccessful. We propose three potential reasons for this: (1) The concentration of PrPSc in the blood of patients with sHPD may have been minuscule, rendering its detection with our current technology unfeasible; (2) PSAs may not be detectable in the blood of these patients; (3) Although our eQuIC assay was shown to be highly sensitive for brain tissue detection, the antibodies used may not be well-suited for blood-based detection. Based on insights from past research, we consider all three hypotheses to account for our findings. However, we believe the combined likelihood of the first and second hypotheses to be the most plausible explanation. Hence, we recognize the importance of exploring alternative detection methods beyond eQuIC for measuring prion activity from blood samples of patients with sHPD.

## Conclusions

In conclusion, although we successfully developed a novel method (eQuIC) that is approximately 100 times more sensitive than the previously used amplification methods, its application for blood-based PSA detection remains inconclusive.

Researchers have reported a promising method for detecting prion activity in tears [14], offering a non-invasive and safer alternative to blood-based testing. We are actively investigating this novel approach, focusing on its advantages in prion detection accuracy and patient safety compared to traditional blood sampling. While many institutions have readily available blood samples, such as plasma or serum, offering a convenient source for past data, tears may hold greater clinical significance in prion detection and tear-based testing might provide more valuable insights for diagnosis and monitoring.

Therefore, new methods must be explored to detect prion activity from the blood of patients with prion diseases.

### Electronic supplementary material

Below is the link to the electronic supplementary material.

Supplementary Material 1

## Data Availability

Data sharing does not apply to this article, as no datasets were generated during the current study.
