# Supplementary material for: Detection limitations of prion seeding activities in blood samples from patients with sporadic prion disease
Source: BMC Neurol. 2024 Mar 11;24:92. doi: 10.1186/s12883-024-03590-7 (PMC10926576; doi:10.1186/s12883-024-03590-7)
Supplement: Supplementary file 1 — Supplementary Material 1 [file 12883_2024_3590_MOESM1_ESM.pdf]

Supplementary Appendix

This appendix provides readers with additional information about this study.

Table of Contents

Supplementary Material P3

Supplementary Table 1 P5

Supplementary Material 1: Supplementary Material and protocols for the second-generation QuIC assay

Production of the Hamster Truncated Recombinant Prion Protein (rHaTrPrP) 90-231

The rHaTrPrP was produced in-house using published protocols as guidelines [1]. More specifically, high-copy number plasmid PRSET (Invitrogen®, USA) was commercially ligated with DNA sequences corresponding to the prion protein (*PRNP)* gene sequence encoding amino acids from 90 to 231 in the hamster. Ligation was performed to strategically bypass the N-terminal His-tag of the plasmid. Chemically competent cells were transformed, and appropriate colonies were selected for glycerol stocks. A tip of glycerol stock was used to inoculate 5 mL LB medium, and the culture was incubated at 37 °C for 5 h with agitation at 220 rpm. The cultures in the LB medium were diluted 1:500 in 4 × 500 mL auto-induction TB medium in non-baffled flasks and incubated at 37 °C overnight with shaking at 200 rpm. Cells were pelleted at 3200 × *g* for 10 min at RT and divided into six 50-mL tubes. Pellets were washed with 2× BugBuster Mastermix (15 mL/10 g wet cells) using mild homogenization and allowed to gently mix by rotation for 10–30 min at RT. Cells were subsequently pelleted at 13,000 × *g*, and the above-described extraction was repeated twice with 10% BugBuster Mastermix. The resulting white pellets were totally solubilized in denaturing buffer (8 M Gnd-HCl, pH 8; 30 mL/cell pellet) by rotation at 10 rpm for 24 h at 4 °C. A volume of 90 mL (total 180 mL in 50% ethanol suspension) of Ni-NTA Superflow was washed with a denaturing phosphate buffer (6 M Gnd-HCl, 100 mM sodium phosphate, 10 mM Tris, pH 7.8) by rotation at 10 rpm (3 × 10 min) followed by centrifugation at 1500 × *g* and supernatant removal. The resulting slurry was equilibrated with the rHaTrPrP solution and allowed to bind by rotation at 10 rpm for 1 h at RT. The slurry was subsequently pelleted and packed in a suitable column onto an ÄKTA FPLC at 10 mL/min for 10 min. The rHaTrPrP was folded with 100 mM sodium phosphate and 10 mM Tris (pH 7.8) at a 5 mL/min flow over 4 h. The product was eluted with a linear imidazole gradient (100 mM sodium phosphate, 10 mM Tris, 500 mM imidazole, pH 6.0) over 45 min at 6 mL/min and collected as a broad peak between 250–350 mM imidazole. The eluate was filter-sterilized (0.2 µm), whereas the concentration was estimated using a spectrophotometer at 250 mM imidazole and subsequently diluted to 0.8 mg/mL with a 10 mM phosphate buffer (pH 6.5). The eluate was dialyzed extensively against a 10 mM phosphate buffer at a pH of 6.5, and the final product concentration was estimated using a Qubit™ 4 fluorometer via a protein assay. The batch was subsequently aliquoted on ice, flash-frozen with liquid nitrogen, and stored at −80 °C.

For the second-generation QuIC protocol, 15 μL of each sample was added to 85 μL of reaction mix in the dark in black, clear-bottom, 96-well microplates. Samples were tested in quadruplicate together with positive (definite CJD) and negative (non-CJD) controls.

Furthermore, the RT-QuIC reaction mix contained 10 mM phosphate buffer (pH 7.4), 300 mM NaCl, 1 mM ethylenediaminetetraacetic acid tetrasodium salt dihydrate (EDTA; pH 8.0), 10 μM thioflavin-T (ThT), 0.002% of sodium dodecyl sulfate (SDS), and 0.1 mg/mL of the Syrian hamster recombinant truncated form of the prion protein (Ha rPrP 90–231).

After sealing, the plate was incubated in a FLUOstar OMEGA reader (BMG Labtech, Germany) at 55 °C, over a period of 60 h with intermittent cycles of shaking (60 s, 700 rpm, double-orbital) and rest (60 s). The fluorescence intensity of the RT-QuIC reaction, expressed as relative fluorescence units (RFUs), was measured every 45 min at 450 ± 10 nm for excitation and 480 ± 10 nm for emission, with a bottom read and a gain of 1000.

References

1. Wilham, J.M.; Orrú, C.D.; Bessen, R.A.; Atarashi, R.; Sano, K.; Race, B.; Meade-White, K.D.; Taubner, L.M.; Timmes, A.; Caughey, B. Rapid end-point quantitation of prion seeding activity with sensitivity comparable to bioassays. *PLOS Pathog.* **2010**, *6*, e1001217. DOI:10.1371/journal.ppat.1001217.

2. Franceschini, A.; Baiardi, S.; Hughson, A.G.; McKenzie, N.; Moda, F.; Rossi, M.; Capellari, S.; Green, A.; Giaccone, G.; Caughey, B.; et al. High diagnostic value of second generation CSF RT-QuIC across the wide spectrum of CJD prions. *Sci. Rep.* **2017**, *7*, 10655. DOI:10.1038/s41598-017-10922-w.

Supplementary Table 1. Summary of sporadic human prion disease patient blood (serum, plasma, and total blood) analyzed using the eQuIC assay by second generation

Patient number	1	2	3
log SD_{50}/g tissue in brains homogenates based on the eQUIC assay (mean ± SD)	12.5 ± 0.15	12.0 ± 1.50	11.75 ± 0.75
log SD_{50}/g tissue in bloods based on the eQUIC assay	N.D.	N.D.	N.D
